# Ketone Bodies Impact on Hypoxic CO_2_ Retention Protocol During Exercise

**DOI:** 10.3389/fphys.2021.780755

**Published:** 2021-12-13

**Authors:** Philip J. Prins, Jeffrey D. Buxton, Tyler S. McClure, Dominic P. D’Agostino, Dana L. Ault, Gary L. Welton, Dalton W. Jones, Adam D. Atwell, Macey A. Slack, Marah L. Slack, Chloe E. Williams, Morgan E. Blanchflower, Kristia K. Kannel, Madison N. Faulkner, Hannah L. Szmaciasz, Stephanie M. Croll, Lindsey M. Stanforth, Tim D. Harris, Holton C. Gwaltney, Andrew P. Koutnik

**Affiliations:** ^1^Department of Exercise Science, Grove City College, Grove City, PA, United States; ^2^Human Healthspan, Resilience, and Performance, Institute for Human and Machine Cognition, Pensacola, FL, United States; ^3^Department of Molecular Pharmacology and Physiology, University of South Florida, Tampa, FL, United States; ^4^Department of Psychology, Grove City College, Grove City, PA, United States

**Keywords:** ketone bodies, hypoxia, CO_2_, cognition, metabolism, exercise, beta-hydroxybutyrate

## Abstract

Exogenous ketone esters have demonstrated the capacity to increase oxygen availability during acute hypoxic exposure leading to the potential application of their use to mitigate performance declines at high altitudes. Voluntary hypoventilation (VH) with exercise reliably reduces oxygen availability and increases carbon dioxide retention without alterations to ambient pressure or gas content. Utilizing a double-blind randomized crossover design, fifteen recreational male distance runners performed submaximal exercise (4 × 5 min; 70% VO_2_ Max) with VH. An exogenous ketone ester (KME; 573 mg⋅kg^–1^) or iso-caloric flavor matched placebo (PLA) was consumed prior to exercise. Metabolites, blood gases, expired air, heart rate, oxygen saturation, cognition, and perception metrics were collected throughout. KME rapidly elevated *R*-β-hydroxybutyrate and reduced blood glucose without altering lactate production. KME lowered pH, bicarbonate, and total carbon dioxide. VH with exercise significantly reduced blood (SpO_2_) and muscle (SmO_2_) oxygenation and increased cognitive mean reaction time and respiratory rate regardless of condition. KME administration significantly elevated respiratory exchange ratio (RER) at rest and throughout recovery from VH, compared to PLA. Blood carbon dioxide (PCO_2_) retention increased in the PLA condition while decreasing in the KME condition, leading to a significantly lower PCO_2_ value immediately post VH exercise (IPE; *p* = 0.031) and at recovery (*p* = 0.001), independent of respiratory rate. The KME’s ability to rapidly alter metabolism, acid/base balance, CO_2_ retention, and respiratory exchange rate independent of respiratory rate changes at rest, during, and/or following VH exercise protocol illustrates a rapid countermeasure to CO_2_ retention in concert with systemic metabolic changes.

## Introduction

Acute and chronic hypoxia exposure has been demonstrated to induce alterations in cognitive function (McMorris et al., 2017; Jung et al., 2020). As altitude increases, barometric pressure drops, leading to reduced oxygen (O_2_) availability and subsequent lung uptake. Reduced uptake leads to a decline in hemoglobin O_2_ saturation (SpO_2_) affecting systemic and cerebral metabolism. Cognition deficits are observed during moderate declines in systemic O_2_ as circulating O_2_ levels function as a strong predictor of cognitive performance during hypoxia exposure (McMorris et al., 2017). These effects appear dose dependent as performance is weaker at lower circulating O_2_ levels. Endogenous compensatory mechanisms which mitigate cognitive decline in hypoxia include vasoconstriction and increased cerebral blood flow (CBF; Feldman et al., 2013). However, these effects are typically only observed with severe drops in O_2_ partial pressure (60 mmHg) and cognitive decline is observed well before these levels are achieved. Therefore, any intervention or treatment that increases CBF while maintaining a higher circulating O_2_ may mitigate the cognitive decline observed during moderate to low systemic O_2_ concentrations.

Ketone bodies (KBs) [β-hydroxybutyrate (βHB), Acetoacetate (AcAc), & Acetone] are metabolically active substrates that provide the brain and extra-hepatic tissues with an alternative oxidizable fuel source. KBs are produced via hepatic ketogenesis during times of carbohydrate deprivation or via ingestion of exogenous ketones sources such as ketone esters, salts, or fatty acids (Evans et al., 2017; Poff et al., 2020). KBs are potent vasodilators that have been shown to increase CBF by 39% during exogenous sodium βHB infusions (Hasselbalch et al., 1996). Additionally, KBs have often been theorized to hold energetic advantages over carbohydrate as the free energy of ATP hydrolysis (Δ*G*′ATP) is greater with KBs and requires less oxygen per mole of carbon to oxidize (Veech, 2004). This concept has been supported in perfused working rat heart models, demonstrating a 28% increase in hydraulic efficiency when exposed to KBs (Sato et al., 1995; Kashiwaya et al., 1997). The exogenous ketone monoester [KME; (R)-3-hydroxybutyl (R)-3-hydroxybutyrate] has demonstrated the ability to maintain a significantly higher SpO_2_ saturation during severe hypoxic exposures of 20,000ft (Coleman et al., 2021). Moreover, KBs have been demonstrated to be neuroprotective (Maalouf et al., 2009), improving cognitive performance in patients with cognitive impairment (Reger et al., 2004) and brain hypometabolism (Croteau et al., 2018; Fortier et al., 2021), and potentially ameliorating the effects of acute brain injury and ischemic damage (White and Venkatesh, 2011; Hinojo et al., 2021) making KBs an enticing intervention to mitigate cognitive declines observed during acute or chronic hypoxic exposure.

Traditionally hypoxic exposure is achieved through physically traveling to high elevations or altering ambient air and pressure of a room to mimic high altitude conditions (Ando et al., 2020). Recent research has used alterations in breathing patterns called voluntary hypoventilation (VH) coupled with moderate to intense exercise to elicit a similar drop in SpO_2_ (< 95%, clinically hypoxic) and rise in the partial pressure of carbon dioxide (PCO_2_). VH limits both O_2_ inspiration and CO_2_ expiration which creates a hypoxia setting as well as a build-up of CO_2_ in circulation which can eventually lead to hypercapnia (Woorons et al., 2010, 2011).

**FIGURE 1:**
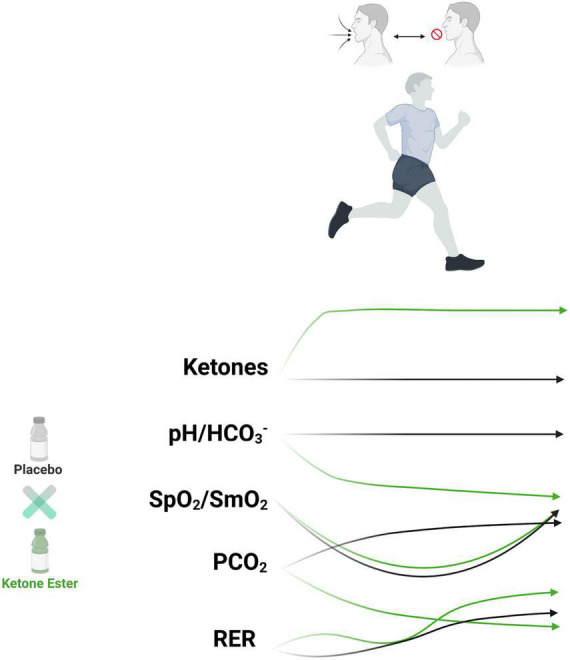
Exogenous ketone bodies metabolic and physiologic impact on hypoxia and carbon dioxide retention exercise protocol. Runners (*n* = 15) undergoing breath hold exercise protocol had reduced systemic and muscular oxygen saturation (hypoxemia) with increased carbon dioxide retention. Exogenous ketone bodies increased circulating ketone bodies, increased metabolic acid load, reduced carbon dioxide retention, elevated respiratory exchange rate, amongst other effects. HCO_3_^–^, bicarbonate; PCO_2_, partial pressure of carbon dioxide; pH, power of hydrogen; RER, respiratory exchange rate; SpO_2_, systemic oxygen saturation; SmO_2_, muscle oxygen saturation.

Therefore, the main goal of this present study was to investigate the effects of KME ingestion on blood gas concentrations through alterations in metabolism and pH at rest, during exercise, and recovery. The second objective was to determine how KME ingestion affected cognitive performance before and after a hypoxia-inducing exercise protocol. We elucidated unique metabolic, blood gas and gas exchange alterations with ketone administration utilizing a VH protocol (Figure 1).

## Materials and Methods

### Study Design

**FIGURE 2:**
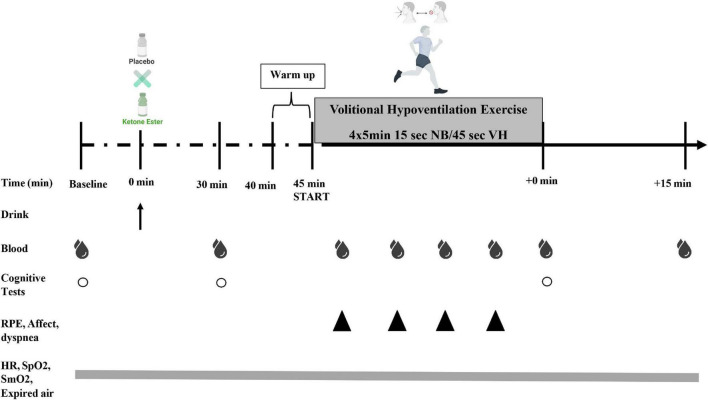
Study experimental design. Subjects consumed (R)-3-hydroxybutyrate ketone monoester (KME) mixed with carbohydrate solution or ketone-free, isocaloric, and flavor matched placebo (PLA) beverage. Capillary metabolites and blood gases were assessed pre-drink (PRE), 30 min post-drink (30 min), during the volitional hypoventilation protocol (VH; end of each set; blood gases only at end of VH set#2), immediately following the VH exercise (IPE), and 15 min post VH exercise protocol (+15 min). Subjective ratings of perceived exertion (RPE), affect, and dyspnea were measured during the VH (end of each set; VH set#1–4). A cognitive performance testing battery was be performed at PRE, 30 min, and IPE (COG 1–3). Heart rate (HR), arterial oxygen saturation (SpO_2_) muscle tissue oxygenation (SmO_2_), and respiratory gases [oxygen consumption [VO_2_], carbon dioxide production [VCO_2_], pulmonary ventilation [VE], and respiratory exchange ratio (RER)] was measured continuously throughout protocol.

A randomized^1^, double-blind, placebo-controlled, and cross-over design was employed consisting of four separate laboratory visits (Figure 2). During visit 1 each subject underwent an informed consent and familiarization. During visit 2 each participant’s maximal oxygen consumption (VO_2_ Max) was determined. Visit 3 and 4 comprised of the VH protocol along with metabolites, heart rate, blood gases, gas exchange, perception, and cognition measurements throughout. During experimental days, subjects consumed either a ketone monoester supplement (KME) or a calorie, flavor, volume, and appearance matched placebo (PLA). Testing sessions were conducted at the Exercise Science Laboratory of Grove City College at the same time each day in a room consistently between 19 and 21°C and 35–40% humidity.

### Participants

Fifteen recreational male distance runners participated. Participants were included if they: (1) ran 3–4 time (16–48 km) per week at moderate to high intensity for ≥ 1 year, (2) were 18 to 35 years of age, and (3) consumed a standard American diet (Shan et al., 2019). Participants were excluded if they had (1) history of smoking, (2) metabolic or cardiovascular disease, (3) orthopedic, musculoskeletal, neurological, psychiatric disorder, and/or any medical conditions that prohibit exercise, (4) habitual prescription medications, (5) exogenous ketone supplements or following a low-carbohydrate or ketogenic diet, (6) ergogenic aids within 1 month of study participation. Participants were instructed to refrain from caffeine and alcohol consumption for 48 h and racing or training for 24 h, and food and drink for 3 h (post-prandial minimum time window) before each experimental trail. Participants were instructed to maintain their usual training frequency and log (mode, duration, and intensity of each workout) throughout the study intervention (Prins et al., 2020). Training load for each session was calculated by using the session-RPE method (RPE × session duration [min]) (Foster et al., 2001). Furthermore, participants’ habitual pre-trial and within trial dietary intake was assessed weekly using a 3-day weighed dietary record, consisting of 2 weekdays and 1 weekend day (Nutritionist Pro™, Axxya Systems, Stafford, TX, United States). Participants were provided with a copy of their pre-trial log and instructed to have the same dietary intake during the remainder of the study (Prins et al., 2020). No significant differences were observed in nutrient intake (Supplementary Table 1) and training load (Supplementary Table 2). The experimental protocol was approved by the Institutional Review Board of Grove City College.

### Visit 1: Familiarization and Anthropometrics

Subjects underwent an orientation involving practice of the intermittent VH, treadmill running and familiarization of the various measurement instruments (i.e., cognitive test battery), equipment, and subjective measurement scales. Following the orientation session, height (cm; Detecto, Webb City, MO, United States), weight (kg), fat free mass (kg), and fat mass (% and kg) were obtained (Table 1). Weight (kg) and body composition (fat and lean mass) were measured via bioelectrical impedance analyzer (BIA; TBF-310GS Tanita Corporation of America, Arlington Heights, IL, United States).

**TABLE 1:** Participant characteristics.

Variable	Mean ± SD
Age (years)	20.6 ± 2.1
Height (cm)	180.1 ± 7.8
Weight (kg)	76.7 ± 9.6
BMI (kg/m^2^)	23.6 ± 2.9
Body fat (%)	12.8 ± 5.7
Fat mass (kg)	10.0 ± 4.6
FFM (kg)	66.5 ± 8.3
Mean running distance/Week (km)	31.4 ± 22.4
Running experience (years)	7.1 ± 2.9
VO_2_ Max (ml/kg/min)	59.3 ± 6.1

*Participant age, anthropometric, and training status (n = 15). Values are Mean ± SD. BMI, body mass index; FFM, fat-free mass; VO_2_ Max, maximum oxygen consumption.*

### Visit 2: Maximal Aerobic Capacity (VO_2_ Max)

Subjects performed an incremental test to exhaustion on a motorized treadmill (Trackmaster TMX425C treadmill, Newton, KS, United States) utilizing the modified Astrand treadmill protocol (Prins et al., 2020). The treadmill was calibrated before each exercise test according to the manufacturer’s instructions. Oxygen consumption (VO_2_) and carbon dioxide production (VCO_2_) was measured using an automated metabolic analyzer system (TrueOne 2400, ParvoMedics, Sandy, UT, United States) calibrated prior to each exercise test using standard calibration gases (16% O_2_ and 4% CO_2_). Heart rate was measured throughout (Polar Electro, Kempele, Finland). The following criteria were used to ensure a physiologically valid VO_2_ Max was attained: (1) a plateau in VO_2_ with increasing exercise intensity (<150 ml/min or <2.1 ml/kg/min), (2) a respiratory exchange ratio (RER) of ≥1.1, and (3) volitional termination due to exhaustion.

### Visit 3 and 4: Experimental Sessions

#### Supplementation

Starting 45 min prior to the VH, participants were allowed 5 min to ingest either the KME or PLA beverage. The KME drink consisted of 573 mg.kg^–1^ body mass of a (R)-3-hydroxybutyl (R)-3-hydroxybutyrate ketone monoester (120 kcal/25g; Pure ΔG Ketone Ester HVMN™ Ketone; HVMN, Inc., San Francisco, CA, United States). The commercially available KME was mixed directly with a carbohydrate solution (60 g of dextrose). The PLA beverage was a ketone-free, isocaloric, flavor matched (0 kcal; HVNM) placebo that contained 668 mg.kg^–1^ body mass of maltodextrin with 60g dextrose. The PLA drink was taste matched using a bitterness additive (Bittrex, Portland, United States). Both drinks were dissolved in water to 500 ml measured with a graduated cylinder. All dry ingredients were measured to the nearest 0.001 g on a calibrated balance scale (Denver Instrument, Bohemia, NY, United States). Participants wore a nose clip during ingestion of the drinks and were given 20 mL of calorie-free sports drink (Gatorade Zero, Chicago, IL, United States) immediately after ingesting the experimental beverages to remove any lingering flavor. While flavor matching has been internally piloted and validated, there remains the possibility that the participants were able to identify the drinks. Thus, to ensure a double-blinded design, each drink was presented to participants in an opaque sports bottle.

Once the experimental protocol was completed, but before participants were informed of the values assessed during each condition, participants were asked, “Please identify which trial the ketone drink was consumed, and which trial was the placebo drink consumed.” When presented between two choices, participants had a 40:60 ratio of incorrect to correct guesses suggesting participant were largely unable to readily detect treatment versus placebo outside of random chance (blinded). After completion of all conditions, participants were thoroughly debriefed.

#### Voluntary Hypoventilation Protocol

The intermittent VH involved submaximal treadmill running while utilizing a VH at low lung volume breathing technique (Woorons et al., 2008). The VH technique involves 15 s of normal unrestricted breathing (NB) followed by 45 s of repeat bouts of an inhalation then exhalation nearing functional residual capacity followed by a 4-s breath hold. Subjects began the protocol with a 5-min warmup at an intensity between 50 and 65% of VO_2_ Max using NB. Following the warmup subjects performed 4 × 5-min sets of running at an intensity of 70% of their VO_2_ Max utilizing VH with 1-min of NB between sets. Previous studies using similar protocols during exercise have demonstrated that this breathing technique results in severe hypoxemia (SpO_2_ < 88%) like hypoxic states obtained at altitudes greater than 2000 m, combined with elevations in circulating CO_2_ levels (CO_2_ retention; Woorons et al., 2008, 2010, 2011, 2017; Fornasier-Santos et al., 2018).

#### Metabolites and Blood Gas

Finger capillary blood lactate (Lactate Plus, Nova Biomedical), ketones (β-hydroxybutyrate; Precision Xtra, Abbott Diabetes Care Inc., Almeda, CA, United States) and glucose (Precision Xtra, Abbott Diabetes Care Inc., Almeda, CA, United States) concentrations were measured. Additional fingertip capillary whole blood droplets were collected within 100 μl capillary tubes and then transferred to a disposable CG4 + cartridge (Abbott Point of Care Inc., Princeton, NJ, United States) and then analyzed for blood pH, HCO_3_^–^, PO_2_, PCO_2_, SaO_2_, and TCO_2_ with the i-STAT analyzer (Abbott Point of Care Inc., Princeton, NJ, United States), which was calibrated prior to each session in accordance with manufacturer’s guidelines (Sediame et al., 1999). Fingertip blood samples were collected using a lancet following alcohol cleaning. The first droplet was wiped away with a cotton swab and the subsequent droplets were used for analysis. Blood samples were measured before supplementation (baseline; PRE), 30 min after supplementation (30 min), at the end of each set of the VH (VH Set 1–4; blood gases only at end of VH Set 2), immediately post-exercise (IPE), and 15 min after exercise (+ 15 min; recovery).

#### Oxygen Saturation, Respiratory Gas, and Heart Rate

Heart rate (HR), systemic oxygen saturation (SpO_2_), muscle tissue oxygenation (SmO_2_), and respiratory gases were measured continuously throughout trials. SpO_2_ was estimated with the Nonin WristOx2™ pulse oximeter (Model 3150, Plymouth, MN, United States), attached to the index finger of the non-dominant hand (Buekers et al., 2018; Fornasier-Santos et al., 2018). The WristOx2 3150 model has an accuracy of ±2% for SpO_2_ measurements (Nonin Medical, 2017). SmO_2_ was measured on the right vastus lateralis (VL) muscle using a validated wireless near-infrared spectroscopy (NIRS) device (Moxy Muscle Oxygen Sensor; Moxy Monitor, Hutchinson, MN, United States; Crum et al., 2017; Piucco et al., 2018) which emits light ranging from 680 to 800 nm. SmO_2_ represents the percentage of total oxygen carrying hemoglobin in the capillaries of the muscle and determined using the following equation:

(Oxygenated hemoglobin + oxygenated myoglobin)/(total hemoglobin + myoglobin) × 100

Gas exchange was recorded using a portable metabolic analyzer (COSMED K5, Rome, Italy). Prior to each experimental session, device was calibrated using procedures according to manufacturer instructions. The breath-by-breath measurements were performed for minute ventilation (VE), oxygen uptake (VO_2_), carbon dioxide production (VCO_2_), and respiratory exchange ratio (RER) and was measured continuously throughout trials. The reported values for HR, SpO_2_, SmO_2_, and respiratory gases represent the overall average over 5 min baseline/PRE, 30 min, IPE, + 15 min. Additionally, aforementioned data were averaged for the entirety of each cognitive test battery (Cog 1–3) and for each set of the VH (VH Set 1–4).

#### Cognition and Perception

At PRE, 30 min, and IPE, participants performed Cog 1, 2, and 3 to assess executive cognitive function using a self-directed computerized automated neuropsychological assessment metric (ANAM^®^) test with established test-retest reliability (ANAM-4, Vista Life Sciences, United States) as previously described (Cernich et al., 2007; Prins et al., 2020). A familiarization test was performed during the first laboratory visit to reduce the possibility of a learning effect. All tests were performed in sound-insulated room under controlled conditions (i.e., appropriate lighting, as quiet as possible, and isolation from unnecessary stimuli and timing feedback). Participants were instructed to complete the battery as quickly and accurately as possible. Each trial was administered identically with reaction time (in milliseconds) and reaction time for correct responses only (accuracy) collected.

Subjective scores of RPE (OMNI Walk/Run Perceived Exertion; Robertson et al., 2004), Affect (feeling scale; Hardy and Rejeski, 1989), and dyspnea (Modified Borg scale; Bausewein et al., 2007) were assessed at the end of each VH set (VH Set 1–4). Gastrointestinal discomfort was rated 5 min after the completion of each experimental trial by means of a validated 0–8 Liker scale questionnaire (Pfeiffer et al., 2009).

### Statistical Analysis

Statistical analyses were performed using SPSS version 26.0 (SPSS Inc., Chicago, IL, United States). Statistical significance was set *a priori* at *p* < 0.05. Descriptive statistics were calculated for all variables. Normality and absence outliers were verified by using the Shapiro–Wilk test, normality plots, and residual plots. Two-way (time-condition) repeated-measures ANOVA was used to determine differences between the two experimental trials for all variables with serial measurements. A one-way repeated-measures ANOVA was used to analyze differences over time. *Post hoc* analyses of significant main effects were conducted where appropriate using the Bonferonni adjustment to determine which conditions were significantly different. The assumption of sphericity was confirmed using Mauchly’s test. Green-house–Geisser epsilon corrections were used when the sphericity assumption was violated. Gastrointestinal symptoms were analyzed using a paired sample *t*-test. Partial-eta squared (η^2^p) was used to report effect size. All data are reported as Mean ± SD.

## Results

### Blood Metabolites

**FIGURE 3:**
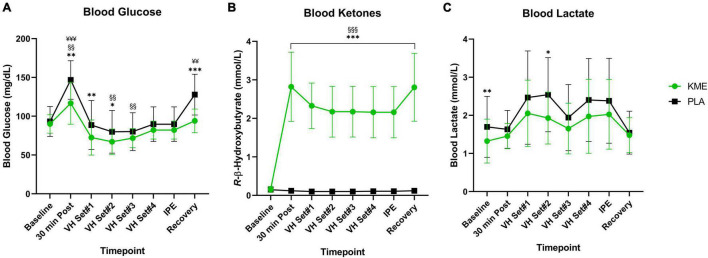
Metabolites. **(A)** Blood glucose, **(B)** *R-*β-hydroxybutyrate, and **(C)** lactate throughout baseline, voluntary hypoventilation exercise protocol (VH), immediately post-exercise (IPE), and recovery with exogenous ketone monoester (KME) or calorie-controlled placebo (PLA). *n* = 15. Data: Mean ± SD. *p<0.05, **p<0.01, ***p<0.001, significant difference between KME and PLA; ^§§^*p*<0.01, ^§§§^*p*<0.001, significant difference between baseline and post-baseline timepoint in KME group. ^¥¥^*p*<0.01, ^¥¥¥^*p*<0.001, significant difference between baseline and post-baseline timepoint in PLA group.

Fasting plasma concentrations of βHB (KME: 0.16 ± 0.07 mM; PLA: 0.15 ± 0.06 mM) and glucose (KME: 90.0 ± 11.9 mg/DL; PLA: 93.3 ± 19.3 mg/dL) did not differ between trials at baseline (Figure 3 and Supplementary Table 3). However, fasting lactate concentrations were significantly lower at baseline compared to PLA (KME: 1.32 ± 0.57 mM; PLA 1.70 ± 0.80 mM; *p* = 0.002). A main effect of time and condition (both *p* < 0.001), as well as time-condition interaction effect (*p* < 0.001) were observed for plasma *R*-βHB concentrations. Blood *R*-βHB increased significantly from baseline to 30 min post in KME condition and remained significantly elevated throughout the trial compared to PLA (*P* < 0.001). Ingestion of KME resulted in a rise of circulating *R*-βHB concentrations to 2.82 ± 0.90 mM (*p* < 0.001) by the start of exercise. These concentrations maintained a 2–3 mM range throughout the KME trial. A main effect for time (*p* = 0.003) and condition (*p* < 0.001), and time-condition interaction effect (*p* < 0.001) were observed for blood glucose concentrations. Blood glucose was significantly lower in KME condition at 30min post exercise, VH Set 1, VH Set 2, and during recovery compared to PLA. Blood glucose was significantly elevated 30 min post, and significantly reduced at VH Set 2 and VH Set 3 timepoints compared to baseline in KME condition. While blood glucose was only significant elevated at the 30 min post and recovery timepoints compared to baseline in the PLA condition. A main effect of time (*p* < 0.001) and condition (*p* = 0.003) were observed for blood lactate. Blood lactate was significantly lower at baseline (*p* = 0.009) and VH Set 2 (*p* = 0.010) compared to PLA. Due to baseline differences in lactate, the change in lactate concentrations from baseline were calculated. No differences were observed in the change in lactate concentration from baseline across groups (Supplementary Figure 1). However, significant time effect was observed. Changes in blood lactate were observed at VH Set 1–2, 4, and IPE compared to 30 min post in PLA condition. While change in blood lactate was only elevated at VH Set 1 in KME condition.

### Oxygen Saturation and Blood Gases

**FIGURE 4:**
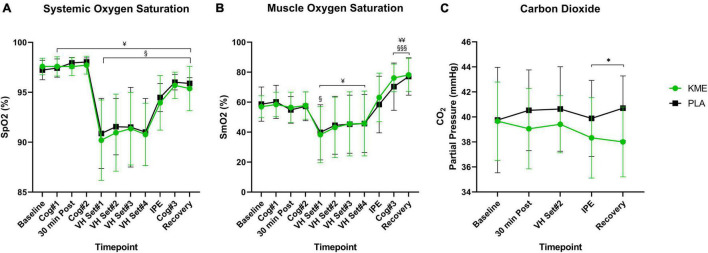
Oxygen saturation and carbon dioxide partial pressure. **(A)** Systemic oxygen saturation, **(B)** muscle oxygen saturation from baseline throughout recovery, **(C)** carbon dioxide throughout baseline, 30 min post, voluntary hypoventilation (VH) set #2, immediately post-exercise (IPE), and recovery with exogenous ketone monoester (KME) or calorie-controlled placebo (PLA). *p<0.05, significant difference between KME and PLA; ^§^*p*<0.05, ^§§§^*p*<0.001, significant difference between baseline and post-baseline timepoint in KME group. ^¥^*p*<0.05, ^¥¥^*p*<0.01 significant difference between baseline and post-baseline timepoint in PLA group.

For both SpO_2_% and SmO_2_%, main effects of time were observed (both *p* < 0.001), but no main effect of condition or time-condition interaction effect were observed (Figure 4). Regardless of condition, SpO_2_% and SmO_2_% significantly decreased during exercise and increased during recovery. SpO_2_% was significantly reduced from VH Set 1 to Recovery in the KME condition compared to baseline. SpO_2_% was significantly elevated compared to baseline prior to VH, and significantly reduced compared to baseline from VH Set 1 to Recovery for PLA condition. SmO_2_% was significantly reduced at VH Set 1 and increased at Cog 3 and Recovery timepoints compared to baseline in the KME condition. Neither a main effect of condition nor a time-condition interaction effect was present for blood PO_2_ (*p* = 0.464; *p* = 0.932, respectively). Conversely, a main effect of condition (*p* = 0.011) and time-condition interaction (*p* = 0.045) were present for blood PCO_2_ (Figure 4 and Supplementary Table 4). Concentrations of blood PCO_2_ were similar at baseline for both conditions until 30 min post supplement ingestion. Thereafter blood PCO_2_ was significantly lower at IPE (*p* = 0.031) and recovery (*p* = 0.001) during the KME condition.

**FIGURE 5:**
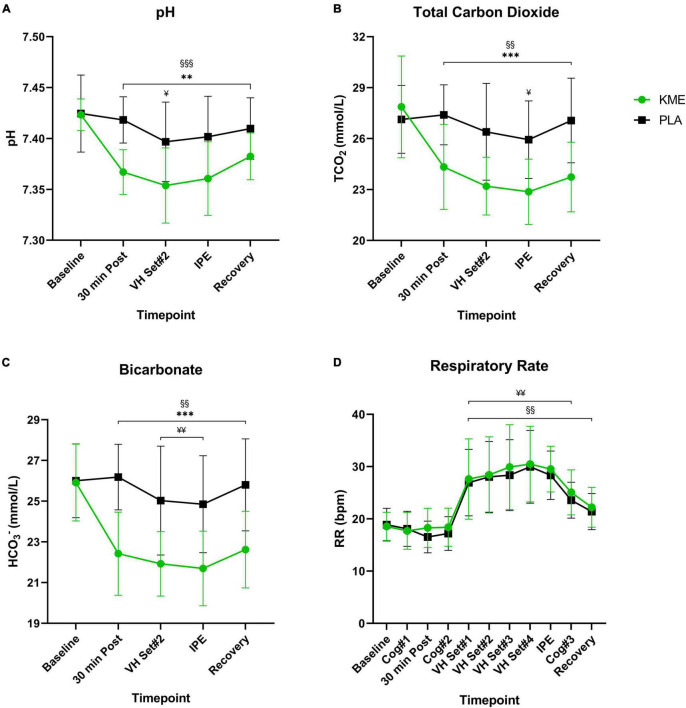
Acid-base balance and respiration rate. **(A)** pH, **(B)** total carbon dioxide, and **(C)** bicarbonate throughout baseline, 30 min post, voluntary hypoventilation (VH) set #2, immediately post-exercise (IPE), and recovery with exogenous ketone monoester (KME) or calorie-controlled placebo (PLA). **(D)** Respiration rate throughout the entire protocol (from baseline to recovery). **p<0.01, ***p<0.001, significant difference between KME and PLA; ^§§^*p*<0.01, ^§§§^*p*<0.001, significant difference between baseline and post-baseline timepoint in KME group. ^¥^*p*<0.05, ^¥¥^*p*<0.01, significant difference between baseline and post-baseline timepoint in PLA group.

For both TCO_2_ and HCO_3_^–^, main effects of time (both *p* < 0.001) and condition (both *p* < 0.001) and time-condition interaction (both *p* < 0.001) were observed (Figure 5 and Supplementary Table 4). Both TCO_2_ and HCO_3_^–^ where significantly lower in KME condition compared to PLA at 30 min post, VH Set 2, IPE, and recovery (*p* < 0.001). TCO_2_ and HCO_3_^–^, TCO_2_ was significantly lower at all post-baseline timepoints compared to baseline in the KME condition. HCO_3_^–^ was significantly lower at VH Set 2 and IPE compared to baseline in the PLA condition. A main effect of time and condition and time-condition interaction (all *p* < 0.001) were observed for blood pH. Blood pH was significantly lower in KME condition compared to PLA at all post baseline timepoints (*p* < 0.001). pH was significantly lower at all post-baseline timepoints compared to baseline in the KME condition. pH was significantly lower at only VH Set 2 compared to baseline in the PLA condition. No main effects for time (*p* = 0.259), condition (*p* = 0.215) and time-condition interaction (*p* = 0.692) were observed between conditions for SaO_2_%.

### Gas Exchange and Heart Rate

**FIGURE 6:**
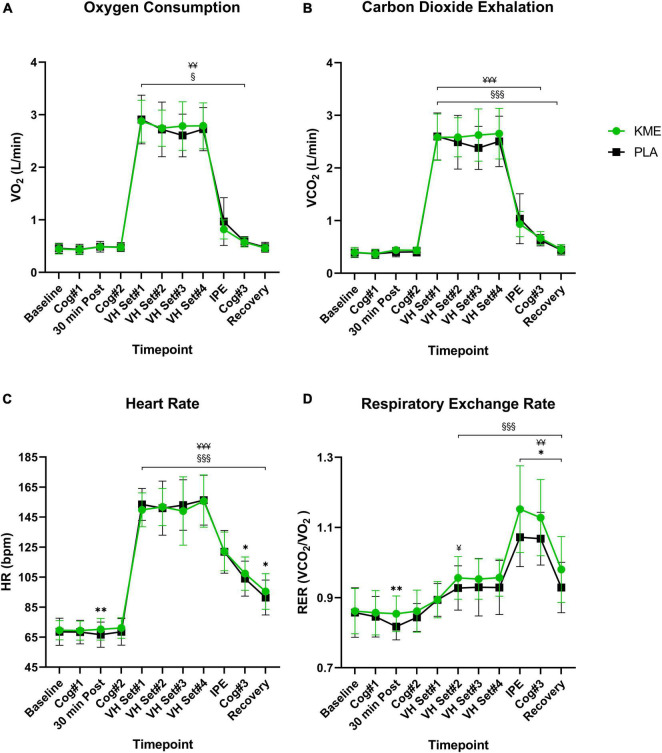
Gas exchange and heart rate. **(A)** Oxygen consumption, **(B)** exhaled carbon dioxide, **(C)** heart rate, and **(D)** respiratory exchange ratio throughout the entire protocol (from baseline to recovery). *p<0.05, **p<0.01, significant difference between exogenous ketone monoester (KME) and calorie-controlled placebo (PLA); ^§^*p*<0.05, ^§§§^*p*<0.001, significant difference between baseline and post-baseline timepoint in KME group. ^¥^*p*<0.05, ^¥¥^*p*<0.01, ^¥¥¥^*p*<0.001, significant difference between baseline and post-baseline timepoint in PLA group. VH, voluntary hypoventilation; IPE, immediately post-exercise.

For VO_2_ and VCO_2_, only a main effect of time was observed (*p* < 0.001; Figure 6 and Supplementary Table 5). No main effects for condition (*p* = 0.927, *p* = 0.129; respectively) or time-condition interaction (*p* = 0.304, *p* = 0.089; respectively) were observed for VO_2_ and VCO_2_. Regardless of the condition, VO_2_ and VCO_2_ remained stable from baseline to Cog 2 (i.e., rest), then increased significantly from Cog 2 to VH Set 1, and then remained significantly elevated during exercise. A main effect of time (*p* < 0.001) and condition (*p* = 0.021) and time-condition interaction (*p* = 0.002) were observed for RER. During the PLA trial, RER was significantly lower 30 min post (*p* = 0.003), IPE (*p* = 0.003), Cog 3 (*p* = 0.020), and at recovery (*p* = 0.030) compared to KME condition. RER was significantly elevated from baseline in KME starting at VH Set 2 and sustained until recovery. RER was significantly elevated from baseline in PLA condition at VH Set 2, IPE, Cog 3, and Recovery timepoints. There was a main effect of time (*p* < 0.001) and condition (*p* = 0.043), but no time-condition interaction (*p* = 0.180) for VE. The trend was for ventilation during exercise and Cog 3 to be significantly higher (VH Set 3 and Cog 3: *p* = 0.029) for KME compared to PLA. While a main effect of time (*p* < 0.001) and condition (*p* = 0.046) effect were observed for respiratory rate (RR), no time by interaction effect was observed. However, RR was elevated from VH Set 1 to recovery in the KME condition compared to baseline, while it was elevated from VH Set 1 to Cog 3 in the PLA condition compared to baseline. For VO_2_ (ml/kg/min), only a main effect of time was observed (*P* < 0.001). No main effect for condition (*p* = 0.512) or time-condition interaction (*p* = 0.195) were observed for VO_2_ (Supplementary Table 5). Regardless of the condition, relative VO_2_ remained stable from baseline to Cog 2 (i.e., rest), then increased significantly from Cog 2 to VH Set 1 and remained significantly elevated during exercise.

A main effect of time (*P* < 0.001) and time-condition interaction (*p* = 0.010) were observed for HR (Figure 6 and Supplementary Table 5). There was a trend for HR in the KME condition to be significantly elevated compared to PLA at 30 min (*p* = 0.008), Cog 3 (*p* = 0.011), and recovery (*p* = 0.017) timepoints, with similar HR responses during exercise. HR remained elevated in both conditions following Cog 2 compared to baseline.

### Cognition and Perception

A main effect of time was observed for Stroop congruent-MRT (*p* = 0.031) and MRT correct (*p* = 0.047 and Supplementary Table 6). Furthermore, both measurements, regardless of condition, improved significantly (i.e., got faster) from baseline values. Similarly, a main effect of time was observed for Stroop incongruent-MRT (*p* < 0.001). This value followed a similar trend of increasing in speed as the trial progressed regardless of condition. No significant effect was observed for Stroop incongruent-MRT correct responses (*p* = 0.052). There was a main effect of time observed for switching-MRT and MRT correct (both *p* = 0.002). Moreover, these cognitive measures followed the same trend of increasing speed (reaction time) from baseline, as noted in the other cognitive evaluations.

For both RPE and dyspnea, a main effect of time was observed (*p* < 0.001; Supplementary Table 5). RPE and dyspnea, regardless of condition, significantly increased over time (i.e., during exercise). For Affect, a main effect of time was observed (*p* < 0.001) and, regardless of condition, significantly decreased during exercise. No significant differences were observed in gastrointestinal effects (*p* = 0.583; Supplementary Table 7) throughout the study.

## Discussion

The present study investigated whether the acute ingestion of a KME supplement altered metabolism, blood gases, gas exchange, cognitive function and perception pre-, during, and post- a hypoxic CO_2_ retention utilizing VH exercise protocol (Figures 1, 2). The VH exercise protocol successfully reduced systemic and muscle oxygen saturation while increasing CO_2_ retention. Compared with PLA, ingestion of KME elevated plasma R-βHB to ∼2.8 mM and significantly attenuated the rise in blood glucose (∼26%), without significantly altering ΔLactate between trials. Consistent with prior reports, KME significantly reduced pH, TCO_2_, and HCO_3_^–^ throughout. KME did not alter the cognitive performance or significantly affect O_2_ availability or saturation compared to PLA with VH. Interestingly, KME blunted the rise in PCO_2_ while significantly increasing RER and HR 30 min post-ingestion compared to PLA. Both PCO_2_ and RER remained significantly altered at all post-VH timepoints compared to PLA without significant alterations in VO_2_, VCO_2_, or RR. Similarly, while VCO_2_ and VO_2_ were unchanged, RER was significantly higher at all post-VH timepoints. Together these results illustrate novel alterations in blood, inspiration, and expiration gas biomarkers both at rest and in response to VH exercise protocol by exogenous KB administration.

The present study adds to the growing body of literature investigating the effects of exogenous KBs to alter metabolism via increasing KB availability while attenuating a rise in blood glucose concentration. All of which have been demonstrated in exogenous ketone esters [KME; and 1,3-butanediol acetoacetate diester (KDE)] (Cox et al., 2016; Leckey et al., 2017; Stubbs et al., 2017; Dearlove et al., 2019) and ketone salts (O’Malley et al., 2017; Stubbs et al., 2017; Evans et al., 2018). The ability of exogenous KBs to decrease glucose concentration is often associated with a decline in lactate production, a relationship that has been observed throughout most exogenous ketone ester research (Cox et al., 2016; Leckey et al., 2017; Evans and Egan, 2018; Dearlove et al., 2019, 2021; Poffé et al., 2020). However, the current investigation and four other studies have noted no such relationship (Rodger et al., 2017; Evans et al., 2018, 2019; Waldman et al., 2019).

A novel finding from this investigation is the ability for KME to lower PCO_2_ without changes in RR (*p* < 0.05). While no one has looked at PCO_2_ during CO_2_ retention protocol, a few other investigations looked at PCO_2_ values from rest through incremental exercise. Dearlove et al. (2019) observed PCO_2_ decreases after ingestion of KME at rest and significant decreases during exercise at stages 100, 150, and 200 W compared to CON (*p* < 0.01, *p* < 0.01, *p* < 0.05; respectively). Additionally, PO_2_ was higher in the KME condition for the entire exercise duration, although no differences in VCO_2_ occurred. Poffé et al. (2020) observed a significant decrease of ∼10 mm Hg in PCO_2_ at the end of exercise due to increase RR. Additionally, VCO_2_ and PO_2_ were elevated without a change in RER. However, we did observe elevations in RER which suggests that CO_2_ expiration may be a partial contributor to the results observed herein and that KME directly modulate RER. This novel ability of KME to blunt PCO_2_ during a CO_2_ retention protocol without changes in RR directly demonstrates ketone-induced alteration in CO_2_ independent of respiratory compensation which could have therapeutic implications for individuals in enclosed, recirculated, undersea, or high intensity exercise environments (Internal Space Station, Submarines, Diving, Aircrafts, etc.) or those experiencing chronic obstructive pulmonary disease (COPD), where elevations in atmospheric CO_2_ and/or the rate CO_2_ production outpaces CO_2_ expiration leads to increased CO_2_ retention. KME could potentially provide a more efficient metabolic substrate that utilizes less O_2_ per carbon oxidized while lowering PCO_2_ before exercise and attenuating the rise during exercise.

Respiratory exchange ratio is a ubiquitous measurement in exercise physiology research used to estimate fuel utilization during exercise and at rest (Péronnet and Massicotte, 1991). However, this method does not account for the metabolism of KBs or the additional VCO_2_ increases related to acute metabolic acidosis, and at present should not be used to describe fat or carbohydrate oxidation shifts. However, despite these limitations, it is still a commonly used measurement in exogenous ketone research where findings have demonstrated mixed results (Cox et al., 2016; Leckey et al., 2017; O’Malley et al., 2017; Rodger et al., 2017; Evans et al., 2018, 2019; Dearlove et al., 2019, 2021; Poffé et al., 2020; Prins et al., 2020). Cox et al. (2016) were the first to utilize RER to determine KB, carbohydrate, and fat oxidation during exercise in exogenous ketosis. They observed a significant decrease in RER upon the start of exercise compared to carbohydrate alone (*p* < 0.05). They reported that KB oxidation accounted for 18% and 16% of O_2_ consumption during exercise in elite cyclists, while oxidizing KBs at a rate of 0.35 g/min and ∼0.5 g/min (40 and 75% Wmax, respectively). These values were determined through indirect calorimetry adjustments for KB oxidation using a method proposed by Frayn (Frayn, 1983). This adjustment quantifies differences in storage and utilization to estimate overall oxidation rates during steady-state exercise. Storage is calculated by multiplying the increase in plasma concentration (including KBs lost in urine and acetone secretion) by the volume of distribution (VD). Frayn applied a VD of 0.2 l/kg of body weight, although VDs as high as 0.5 l/kg have been proposed in more complex four-compartment models (Keller et al., 1981; Cobelli et al., 1982; Frayn, 1983). However, Frayn calculations were not experimentally designed to determine direct KB confirmation in RER and gas exchange parameters. Consequently, utilizing RER to determine fat and carbohydrate oxidation, even when attempting to account for KB contribution using Frayn (1983) data appears unjustified and potentially inaccurate, and thus were not calculated in the present analyses. Notably, very few studies have directly observed RER changes at rest with KB administration. These findings mentioned prior, coupled with the findings of the current investigation, demonstrate the need to reevaluate RER as a tool for understanding fuel utilization during exogenous ketosis as alterations in acid load create the release of additional CO_2_ independent of substrate metabolism.

Cognition improved through each time point of the study, supported by significant increases in reaction time (*p* < 0.05). Potential reasons for this trend are the degree of hypoxia induced by VH and the addition of exercise and the compensatory effect of exercise on cognition in hypoxia environments. Cognitive performance during hypoxia exposure is strongly correlated to SpO_2_ saturation (Ochi et al., 2018). This novel VH protocol induced hypoxia but not to the degree that cognitive declines would be expected. Additionally, VH is a unique acute hypoxia exposure as the limited oxygen availability is not constant like it would be during high elevation or in a high-altitude chamber. VH allows for reoxygenation to occur between breath holds as normal air under ambient pressure enters the lungs (Woorons et al., 2017, 2019). Moreover, this VH protocol utilizes exercise to deplete O_2_ availability further. The confounding nature of exercise is likely to explain both the lack of cognitive decline in hypoxia and/or observations across groups within the current protocol. As exercise itself can act as a potent booster of cognition as it increases CBF and catecholamine release, both of which work to maintain a high metabolic rate of cerebral oxidation (Chang et al., 2012; McMorris et al., 2016) and may represent an overlapping compensatory mechanism to exogenous KBs in the context of hypoxia. VH has been shown to reduce oxygen saturation reliably, although since reoxygenation occurs between breath holds, it does not entirely mimic a real acute hypoxia exposure.

## Conclusion

This study demonstrated the ability of KME to rapidly alter metabolism, blood gas concentrations, and gas exchange during a novel VH exercise protocol. These novel finding opens the door to future therapeutic investigations into enclosed, recirculated, high intensity exercise, and disease states where individuals are at risk for PCO_2_ retention.

## Data Availability Statement

The original contributions presented in the study are included in the article/Supplementary Material, further inquiries can be directed to the corresponding author.

## Ethics Statement

The studies involving human participants were reviewed and approved by Grove City College Institutional Review Board. The patients/participants provided their written informed consent to participate in this study.

## Author Contributions

PP, AK, and JB conceptualized original study design. PP, AK, JB, and DD’A provided input on final study design. PP, JB, DA, GW, DJ, AA, MAS, MLS, CW, MB, KK, MF, HS, SC, LS, TH, and HG contributed to subject recruitment, processing, and data acquisition. PP, AK, and GW conducted statistical analyses. AK and PP developed figures and tables. AK, TM, PP, and JB conducted data interpretation. TM, AK, and PP developed first draft. All authors contributed to the document development, review and contents, and agreed to the accuracy of the work.

## Conflict of Interest

AK is a consultant for Simply Good Foods Inc. DD’A is co-owner of Ketone Technologies LLC, providing scientific consulting and public speaking on ketogenic therapies, and receives royalties in accordance with University of South Florida policy patent policy. The remaining authors declare that the research was conducted in the absence of any commercial or financial relationships that could be construed as a potential conflict of interest.

## Publisher’s Note

All claims expressed in this article are solely those of the authors and do not necessarily represent those of their affiliated organizations, or those of the publisher, the editors and the reviewers. Any product that may be evaluated in this article, or claim that may be made by its manufacturer, is not guaranteed or endorsed by the publisher.

## Footnotes

1
randomizer.org

## Supplementary Material

Supplementary Figure 1 Change in blood lactate. Change in blood lactate was compared to baseline with exogenous ketone monoester (KME) or calorie-controlled placebo (PLA). *n* = 15. Data: Mean ± SD. ^§^*p*<0.05, significant difference between baseline and post-baseline timepoint in KME group. ^¥¥^*p*<0.01, significant difference between baseline and post-baseline timepoint in PLA group. VH, voluntary hypoventilation; IPE, immediately post-exercise.
