# Supplementary material for: Ketone Bodies Impact on Hypoxic CO2 Retention Protocol During Exercise
Source: Front Physiol. 2021 Dec 13;12:780755. doi: 10.3389/fphys.2021.780755 (PMC8711099; doi:10.3389/fphys.2021.780755)
Supplement: Supplementary file 1 [file Data_Sheet_1.docx]

SUPPLEMENTAL TABLE 1. Nutrient intake

| Diet | Pre-Trial | Week 1 | Week 2 | P-Value |
| --- | --- | --- | --- | --- |
| Calories (Kcals) | 2892.5 ± 1074.0 | 2978.5 ± 1086.4 | 2675.0 ± 782.1 | 0.299 |
| Carbohydrate (g) | 310.3 ± 134.1 | 309.2 ± 135.9 | 271.7 ± 87.5 | 0.175 |
| Protein (g) | 147.4 ± 77.2 | 155.9 ± 86.4 | 141.8 ± 73.4 | 0.645 |
| Fat (g) | 116.3 ± 40.2 | 123.4 ± 48.4 | 112.0 ± 32.3 | 0.486 |
| Carbohydrate (%) | 42.6 ± 6.2 | 41.8 ± 7.8 | 41.5 ± 5.8 | 0.721 |
| Protein (%) | 20.2 ± 3.9 | 20.8 ± 6.6 | 19.8 ± 3.8 | 0.777 |
| Fat (%) | 37.2 ± 3.6 | 37.5 ± 3.8 | 38.7 ± 4.5 | 0.219 |
| Cholesterol (mg) | 490.3 ± 283.3 | 551.0 ± 312.1 | 525.4 ± 214.9 | 0.594 |
| Saturated fat (g) | 38.5 ± 13.8 | 41.8 ± 17.6 | 37.2 ± 10.1 | 0.474 |
| Monounsaturated fat (g) | 32.2 ± 15.5 | 30.8 ± 21.9 | 32.2 ± 15.3 | 0.690 |
| Polyunsaturated fat (g) | 19.8 ± 13.4 | 19.3 ± 13.1 | 18.7 ± 11.6 | 0.825 |
| Sugar (g) | 107.4 ± 64.6 | 101.6 ± 73.3 | 96.8 ± 57.7 | 0.347 |

Participants Nutrient Intake (*n* = 15). Values are Mean ± SD.

SUPPLEMENTAL TABLE 2. Training Load

|  | **Pre-Trial** | **Week 1** | **Week 2** | **P-Value** |
| --- | --- | --- | --- | --- |
| **Training Load (RPExmin)** | 2116.8 ± 1126.9 | 2237.7 ± 1572.6 | 2066.1 ± 1126.3 | 0.718 |

Participant Training Load (*n*=15). Values are Mean ± SD. Abbreviations: RPE, rate of perceived exertion.

SUPPLEMENTAL TABLE 3. Blood Metabolites

|  | Time | | | | | | | | |  |
| --- | --- | --- | --- | --- | --- | --- | --- | --- | --- | --- |
|  | **PRE** | **30 min** | **VH Set 1** | **VH Set 2** | **VH Set 3** | **VH Set 4** | **IPE** | **Recovery** | ***P-Value*** | |
| Blood *R*-β-hydroxybutyrate (mmol/L) |  |  |  |  |  |  |  |  |  | |
| KME | 0.16 ± 0.07 | 2.82 ± 0.90 | 2.33 ± 0.59 | 2.17 ± 0.66 | 2.17 ± 0.66 | 2.16 ± 0.67 | 2.16 ± 0.67 | 2.81 ± 0.88 | Time, *P*<0.001; η^2^p, 0.803  Condition, *P*<0.001; η^2^p, 0.931  Interaction, *P*<0.001; η^2^p, 0.814 | |
| PLA | 0.15 ± 0.06 | 0.10 ± 0.00 | 0.10 ± 0.00 | 0.10 ± 0.00 | 0.10 ± 0.00 | 0.11 ± 0.03 | 0.11 ± 0.03 | 0.12 ± 0.04 |  |  |
| Blood Glucose (mg/dl) |  |  |  |  |  |  |  |  |  | |
| KME | 90.0 ± 11.9 | 116.7 ± 27.0 | 72.5 ± 22.6 | 67.1 ± 16.5 | 71.7 ± 12.3 | 82.1 ± 11.6 | 82.1 ± 11.6 | 94.0 ± 15.2 | Time, *P*=0.003; η^2^p, 0.693  Condition, *P*<0.001; η^2^p, 0.490  Interaction, *P*<0.001; η^2^p, 0.294 | |
| PLA | 93.3 ± 19.3 | 146.7 ± 24.9 | 88.6 ± 31.7 | 79.9 ± 27.5 | 80.1 ± 24.4 | 89.7 ± 22.3 | 89.7 ± 22.3 | 127.8 ± 26.3 |  |  |
| Blood Lactate (mmol/L) |  |  |  |  |  |  |  |  |  | |
| KME | 1.32 ± 0.57 | 1.46 ± 0.33 | 2.05 ± 0.87 | 1.93 ± 0.68 | 1.65 ± 0.66 | 1.97 ± 0.97 | 2.03 ± 0.91 | 1.48 ± 0.46 | Time, *P*<0.001; η^2^p, 0.294  Condition, *P*=0.003; η^2^p, 0.489  Interaction, *P*= 0.533; η^2^p, 0.059 | |
| PLA | 1.70 ± 0.80 | 1.63 ± 0.50 | 2.47 ± 1.22 | 2.54 ± 0.97 | 1.94 ± 0.87 | 2.40 ± 1.09 | 2.38 ± 1.12 | 1.55 ± 0.56 |  |  |

Finger capillary blood glucose, *R*-β-hydroxybutyrate, and lactate (*n*=15). Values are Mean **±** SD. Abbreviations: 30min, 30 minutes after supplementation; IPE, immediately post-exercise KME, exogenous ketone monoester; PLA, flavored matched control; PRE, baseline before supplementation; Recovery, 15 minutes after exercise; VH, voluntary hypoventilation.

SUPPLEMENTAL TABLE 4. Acid-Base Balance and Blood Gases

|  | Time | | | | | |
| --- | --- | --- | --- | --- | --- | --- |
|  | **PRE** | **30 min** | **VH Set 2** | **IPE** | **Recovery** | ***P-Value*** |
| pH |  |  |  |  |  |  |
| KME | 7.42 ± 0.02 | 7.37 ± 0.02 | 7.35 ± 0.04 | 7.36 ± 0.04 | 7.38 ± 0.02 | Time, *P<*0.001, η^2^p, 0.466  Condition, *P*<0.001, η^2^p, 0.727  Interaction, *P<*0.001, η^2^p, 0.377 |
| PLA | 7.42 ± 0.04 | 7.42 ± 0.02 | 7.40 ± 0.04 | 7.40 ± 0.04 | 7.41 ± 0.03 |  |
| PCO_2_ (mmHg) |  |  |  |  |  |  |
| KME | 39.7 ± 3.1 | 39.1 ± 3.2 | 39.4 ± 2.3 | 38.3 ± 3.2 | 38.0 ± 2.8 | Time, *P*=0.524, η^2^p, 0.055  Condition, *P*=0.011; η^2^p, 0.378  Interaction, *P*=0.045; η^2^p, 0.157 |
| PLA | 39.8 ± 4.2 | 40.5 ± 3.2 | 40.6 ± 3.4 | 39.9 ± 3.0 | 40.7 ± 2.6 |  |
| HCO_3_^-^ (mmol/L) |  |  |  |  |  |  |
| KME | 25.9 ± 1.9 | 22.4 ± 2.0 | 21.9 ± 1.6 | 21.7 ± 1.8 | 22.6 ± 1.9 | Time, *P<*0.001; η^2^p, 0.632  Condition, *P<*0.001; η^2^p, 0.808  Interaction, *P<*0.001; η^2^p, 0.480 |
| PLA | 26.0 ± 1.8 | 26.2 ± 1.6 | 25.0 ± 2.7 | 24.8 ± 2.4 | 25.8 ± 2.3 |  |
| TCO_2_ (mmol/L) |  |  |  |  |  |  |
| KME | 27.9 ± 3.0 | 24.3 ± 2.5 | 23.2 ± 1.7 | 22.9 ± 1.9 | 23.7 ± 2.1 | Time, *P<*0.001; η^2^p, 0.525  Condition, *P<*0.001; η^2^p, 0.694  Interaction, *P<*0.001; η^2^p, 0.446 |
| PLA | 27.1 ± 2.0 | 27.4 ± 1.8 | 26.4 ± 2.8 | 25.9 ± 2.3 | 27.1 ± 2.5 |  |

Acid-Base Balance and Blood Gases (*n*=15). Values are Mean ± SD. Abbreviations: 30min, 30 minutes after supplementation; HCO_3_, bicarbonate; IPE, immediately post-exercise; KME, exogenous ketone monoester; PCO2, partial pressure of carbon dioxide; pH, power of hydrogen; PLA, flavored matched control; PRE, baseline before supplementation; Recovery, 15 minutes after exercise; TCO_2_, total carbon dioxide; VH, voluntary hypoventilation.

SUPPLEMENTAL TABLE 5. Physiological, Metabolic, Respiratory, Heart Rate, and Perceptual Responses

|  | Time | | | | | | | | | | | |
| --- | --- | --- | --- | --- | --- | --- | --- | --- | --- | --- | --- | --- |
|  | **PRE** | **Cog 1** | **30 min** | **Cog 2** | **VH Set 1** | **VH Set 2** | **VH Set 3** | **VH Set 4** | **IPE** | **Cog 3** | **Recovery** | ***P-Value*** |
| VO_2_ (L/min) |  |  |  |  |  |  |  |  |  |  |  |  |
| KME | 0.45 ± 0.10 | 0.43 ± 0.08 | 0.48 ± 0.06 | 0.48 ± 0.07 | 2.88 ± 0.40 | 2.74 ± 0.34 | 2.78 ± 0.46 | 2.79 ± 0.44 | 0.82 ± 0.18 | 0.58 ± 0.09 | 0.46 ± 0.07 | Time, *P<*0.001; η^2^p, 0.973  Condition, *P*=0.927; η^2^p, 0.001  Interaction, *P*=0.304; η^2^p, 0.078 |
| PLA | 0.46 ± 0.10 | 0.44 ± 0.10 | 0.49 ± 0.10 | 0.48 ± 0.09 | 2.91 ± 0.46 | 2.72 ± 0.52 | 2.60 ± 0.41 | 2.72 ± 0.41 | 0.97 ± 0.45 | 0.59 ± 0.09 | 0.48 ± 0.09 |  |
| VCO_2_ (L/min) |  |  |  |  |  |  |  |  |  |  |  |  |
| KME | 0.40 ± 0.09 | 0.37 ± 0.08 | 0.44 ± 0.07 | 0.44 ± 0.06 | 2.58 ± 0.44 | 2.58 ± 0.37 | 2.63 ± 0.48 | 2.65 ± 0.48 | 0.93 ± 0.24 | 0.67 ± 0.12 | 0.46 ± 0.09 | Time, *P<*0.001; η^2^p, 0.966  Condition, *P*=0.129; η^2^p, 0.157  Interaction, *P*=0.089; η^2^p, 0.108 |
| PLA | 0.39 ± 0.10 | 0.37 ± 0.09 | 0.40 ± 0.09 | 0.41 ± 0.08 | 2.60 ± 0.45 | 2.49 ± 0.51 | 2.38 ± 0.41 | 2.51 ± 0.48 | 1.04 ± 0.47 | 0.63 ± 0.11 | 0.44 ± 0.10 |  |
| RER |  |  |  |  |  |  |  |  |  |  |  |  |
| KME | 0.86 ± 0.06 | 0.86 ± 0.06 | 0.85 ± 0.05 | 0.86 ± 0.06 | 0.89 ± 0.05 | 0.96 ± 0.06 | 0.95 ± 0.06 | 0.96 ± 0.05 | 1.15 ± 0.12 | 1.13 ± 0.11 | 0.98 ± 0.09 | Time, *P<*0.001; η^2^p, 0.862  Condition, *P*=0.021; η^2^p, 0.325  Interaction, *P*=0.002; η^2^p, 0.178 |
| PLA | 0.86 ± 0.07 | 0.85 ± 0.06 | 0.82 ± 0.04 | 0.84 ± 0.04 | 0.89 ± 0.05 | 0.93 ± 0.06 | 0.93 ± 0.08 | 0.93 ± 0.08 | 1.07 ± 0.08 | 1.07 ± 0.08 | 0.93 ± 0.07 |  |
| VO_2_ (ml/kg/min) |  |  |  |  |  |  |  |  |  |  |  |  |
| KME | 6.02 ± 1.49 | 5.75 ± 1.32 | 6.42 ± 0.65 | 6.32 ± 0.81 | 38.23 ± 5.26 | 36.30 ± 5.69 | 36.61 ± 4.95 | 36.69 ± 4.64 | 10.65 ± 2.26 | 7.50 ± 1.11 | 6.12 ± 1.11 | Time, *P<*0.001; η^2^p, 0.975  Condition, *P*=0.512; η^2^p, 0.031  Interaction, *P*=0.195; η^2^p, 0.090 |
| PLA | 6.10 ± 0.93 | 5.78 ± 0.79 | 6.42 ± 0.99 | 6.35 ± 0.82 | 38.03 ± 5.02 | 35.50 ± 6.48 | 34.25 ± 5.81 | 35.48 ± 4.86 | 12.47 ± 5.67 | 6.64 ± 0.88 | 6.27 ± 0.81 |  |
| Ve (L/min) |  |  |  |  |  |  |  |  |  |  |  |  |
| KME | 13.6 ± 1.9 | 12.8 ± 1.9 | 14.5 ± 2.7 | 14.7 ± 2.5 | 65.3 ± 8.9 | 65.5 ± 8.4 | 68.0 ± 10.1 | 68.9 ± 10.3 | 30.4 ± 7.7 | 21.6 ± 4.9 | 16.2 ± 3.4 | Time, *P<*0.001; η^2^p, 0.968  Condition, *P*=0.043; η^2^p, 0.261  Interaction, *P*=0.180; η^2^p, 0.092 |
| PLA | 13.7 ± 3.3 | 12.9 ± 2.8 | 13.4 ± 3.1 | 13.6 ± 2.6 | 64.7 ± 10.3 | 63.7 ± 12.2 | 62.4 ± 10.6 | 66.3 ± 11.9 | 31.1 ± 10.1 | 19.8 ± 3.9 | 14.9 ± 3.5 |  |
| RR (breath/min) |  |  |  |  |  |  |  |  |  |  |  |  |
| KME | 18.6 ± 2.7 | 17.7 ± 3.5 | 18.3 ± 3.7 | 18.4 ± 3.6 | 27.6 ± 7.7 | 28.4 ± 7.3 | 29.9 ± 8.1 | 30.5 ± 7.2 | 29.5 ± 4.4 | 25.1 ± 4.3 | 22.2 ± 3.8 | Time, *P<*0.001; η^2^p, 0.642  Condition, *P*=0.046; η^2^p, 0.255  Interaction, *P*=0.589; η^2^p, 0.057 |
| PLA | 18.9 ± 3.1 | 18.1 ± 3.4 | 16.5 ± 3.0 | 17.2 ± 3.2 | 26.9 ± 6.3 | 28.0 ± 6.8 | 28.4 ± 6.8 | 29.9 ± 6.9 | 28.3 ± 4.6 | 23.6 ± 3.4 | 21.4 ± 3.5 |  |
| HR (beats/min |  |  |  |  |  |  |  |  |  |  |  |  |
| KME | 69.6 ± 6.4 | 69.4 ± 6.4 | 70.2 ± 7.2 | 71.1 ± 6.9 | 149.8 ± 11.3 | 151.7 ± 12.3 | 149.0 ± 22.8 | 155.7 ± 17.4 | 122.1 ± 12.7 | 107.3 ± 11.1 | 95.4 ± 11.9 | Time, *P<*0.001; η^2^p, 0.966  Condition, *P*=0.598; η^2^p, 0.020  Interaction, *P*=0.010; η^2^p, 0.149 |
| PLA | 68.6 ± 9.0 | 68.4 ± 7.8 | 66.6 ± 8.4 | 68.5 ± 8.9 | 153.4 ± 10.6 | 150.9 ± 18.0 | 153.0 ± 16.8 | 156.2 ± 16.6 | 121.8 ± 14.2 | 104.0 ± 11.7 | 91.40 ± 11.6 |  |
| SpO_2_ (%) |  |  |  |  |  |  |  |  |  |  |  |  |
| KME | 97.2 ± 1.0 | 97.4 ± 0.9 | 97.9 ± 0.5 | 98.0 ± 0.4 | 90.9 ± 3.5 | 91.6 ± 2.8 | 91.5 ± 4.0 | 91.0 ± 3.4 | 94.5 ± 1.4 | 96.0 ± 0.8 | 95.9 ± 0.6 | Time, *P<*0.001; η^2^p, 0.735  Condition, *P*=0.363; η^2^p, 0.064  Interaction, *P*=0.823; η^2^p, 0.043 |
| PLA | 97.6 ± 0.8 | 97.6 ± 1.0 | 97.6 ± 0.9 | 97.7 ± 0.9 | 90.2 ± 4.0 | 91.0 ± 3.9 | 91.4 ± 3.6 | 90.8 ± 3.1 | 94.0 ± 2.7 | 95.7 ± 1.3 | 95.4 ± 2.2 |  |
| SmO_2_ (%) |  |  |  |  |  |  |  |  |  |  |  |  |
| KME | 57.0 ± 7.3 | 58.4 ± 8.1 | 56.5 ± 10.1 | 57.7 ± 9.1 | 38.3 ± 18.7 | 43.1 ± 20.2 | 45.5 ± 21.4 | 45.8 ± 21.6 | 63.1 ± 16.2 | 76.2 ± 9.3 | 78.2 ± 11.0 | Time, P<0.001; η^2^p, 0.654  Condition, *P=*0.758; η^2^p, 0.007  Interaction, *P*=0.743; η^2^p, 0.046 |
| PLA | 58.7 ± 11.4 | 60.2 ± 11.1 | 54.8 ± 8.9 | 57.2 ± 9.6 | 39.8 ± 18.4 | 44.6 ± 19.3 | 45.3 ± 19.4 | 45.7 ± 19.3 | 58.4 ± 18.9 | 70.3 ± 15.8 | 77.1 ± 12.5 |  |
| RPE |  |  |  |  |  |  |  |  |  |  |  |  |
| KME | - | - | - | - | 4.7 ± 1.8 | 5.4 ± 1.6 | 5.8 ± 1.1 | 6.3 ± 1.3 | - | - | - | Time, *P*<0.001; η^2^p, 0.615  Condition, *P*=0.563; η^2^p, 0.024  Interaction, *P*=0.858; η^2^p, 0.018 |
| PLA | - | - | - | - | 4.3 ± 1.9 | 5.2 ± 1.9 | 5.7 ± 1.7 | 6.1 ± 1.6 | - | - | - |  |
| Affect |  |  |  |  |  |  |  |  |  |  |  |  |
| KME | - | - | - | - | 1.1 ± 1.5 | 0.3 ± 1.6 | -0.1 ± 1.8 | -0.4 ± 1.9 | - | - | - | Time, *P<*0.001; η^2^p, 0.702  Condition, *P*=1.00; η^2^p, 0.000  Interaction, *P*=0.873; η^2^p, 0.016 |
| PLA | - | - | - | - | 1.1 ± 2.2 | 0.3 ± 2.0 | -0.1 ± 2.0 | -0.3 ± 1.7 | - | - | - |  |
| Dyspnea |  |  |  |  |  |  |  |  |  |  |  |  |
| KME | - | - | - | - | 3.1 ± 1.0 | 3.7 ± 1.3 | 4.3 ± 1.5 | 4.8 ±1.6 | - | - | - | Time, *P<*0.001; η^2^p, 0.507  Condition, *P*=0.694; η^2^p, 0.011  Interaction, *P*=0.889; η^2^p, 0.015 |
| PLA | - | - | - | - | 3.3 ± 1.5 | 3.9 ± 1.6 | 4.3 ± 1.8 | 4.7 ± 1.9 | - | - | - |  |

Physiological, Metabolic, Respiratory, Heart Rate, and Perceptual Responses (*n*=15). Values are Mean ± SD. Abbreviations: 30min, 30 minutes after supplementation; HR, heart rate; IPE, immediately post-exercise; KME, exogenous ketone monoester; PLA, flavored matched control; PRE, baseline before supplementation; RER, respiratory exchange ration; RR, respiratory rate; SpO_2_, hemoglobin oxygen saturation; SmO_2_, muscle oxygen saturation; Recovery, 15 minutes after exercise; RPE, rate of perceived exertion; VCO_2_, expired carbon dioxide; Ve, ventilation; VH, voluntary hypoventilation; VO_2_, oxygen consumption.

SUPPLEMENTAL TABLE 6. Cognitive Function Scores.

| **Test Variable** | **KME** | | | **PLA** | | | ***P*-Value** |
| --- | --- | --- | --- | --- | --- | --- | --- |
|  | **PRE** | **30 min** | **IPE** | **PRE** | **30 min** | **IPE** |  |
| **Stroop, Congruent (ms) (N = 15)** | | | | | | | |
| **Mean Reaction Time** | 577.9 ± 138.6 | 548.9 ± 99.7 | 551.3 ± 105.6 | 558.6 ± 128.6 | 540.9 ± 110.5 | 530.8 ± 107.8 | Time, *P*=0.031; η^2^p, 0.219  Condition, *P*=0.237; η^2^p, 0.098  Interaction, *P*=0.883; η^2^p, 0.009 |
| **Mean Reaction Time Correct** | 578.1 ± 140.4 | 550.4 ± 102.1 | 551.5 ± 105.7 | 558.1 ± 128.1 | 539.6 ± 106.1 | 529.6 ± 102.2 | Time, *P*=0.047; η^2^p, 0.197  Condition, *P*=0.202; η^2^p, 0.114  Interaction, *P*=0.908; η^2^p, 0.007 |
| **Stroop, Incongruent (ms) (N = 15)** | | | | | | | |
| **Mean Reaction Time** | 673.6 ± 176.2 | 633.4 ± 143.6 | 629.8 ± 151.9 | 685.5 ± 192.1 | 619.5 ± 140.6 | 610.3 ± 120.4 | Time, *P*<0.001; η^2^p, 0.438  Condition, *P*=0.754; η^2^p, 0.007  Interaction, *P*=0.669; η^2^p, 0.028 |
| **Mean Reaction Time Correct** | 652.4 ± 190.5 | 637.5 ±152.3 | 628.8 ± 152.8 | 672.2 ± 177.5 | 623.3 ± 143.6 | 614.8 ± 134.2 | Time, *P*=0.052; η^2^p, 0.190  Condition, *P*=0.901; η^2^p, 0.001  Interaction, *P*=0.552; η^2^p, 0.042 |
| **Switching, Manikin and Mathematical Processing Test (ms) (N = 15)** | | | | | | | |
| **Mean Reaction Time** | 1540.9 ± 457.4 | 1428.0 ± 369.8 | 1365.8 ± 341.0 | 1493.5 ± 410.9 | 1437.4 ± 395.9 | 1387.7 ± 364.8 | Time, *P*=0.002; η^2^p, 0.363  Condition, *P*=0.943; η^2^p, 0.000  Interaction, *P*=0.581; η^2^p, 0.038 |
| **Mean Reaction Time Correct** | 1521.9 ± 461.0 | 1423.5 ± 375.3 | 1357.4 ± 344.0 | 1497.1 ± 419.2 | 1434.0 ± 398.7 | 1376.5 ± 370.1 | Time, *P*=0.002; η^2^p, 0.351  Condition, *P*=0.983; η^2^p, 0.000  Interaction, *P*=0.814; η^2^p, 0.015 |

Cognitive Function Test. Mean reaction time and number of correct responses were evaluated via Stroop Congruent, Stroop Incongruent, Switching, Manikin and Mathematical Processing Tests (*n*=15). Values are Mean ± SD. Abbreviations: 30min, 30 minutes after supplementation; IPE, immediately post-exercise; KME, Exogenous Ketone Monoester; PLA, Flavored Matched Control; PRE, baseline before supplementation.

SUPPLEMENTAL Table 7. Gastrointestinal Effects

|  | **KME** | **PLA** | **P-Value** |
| --- | --- | --- | --- |
| **Upper GI** | 0.47 ± 0.83 | 0.67 ± 2.32 | 0.710 |
| **Lower GI** | 0.67 ± 1.29 | 0.33 ± 0.82 | 0.353 |
| **Systemic** | 2.73 ± 3.56 | 2.20 ± 2.68 | 0.584 |
| **Total Symptom Load** | 3.87 ± 4.26 | 3.20 ± 4.63 | 0.583 |

Abbreviations: GI, Gastrointestinal.
