# Supplementary figures and images for: Ketone Bodies Impact on Hypoxic CO2 Retention Protocol During Exercise
Source: Front Physiol. 2021 Dec 13;12:780755. doi: 10.3389/fphys.2021.780755 (PMC8711099; doi:10.3389/fphys.2021.780755)

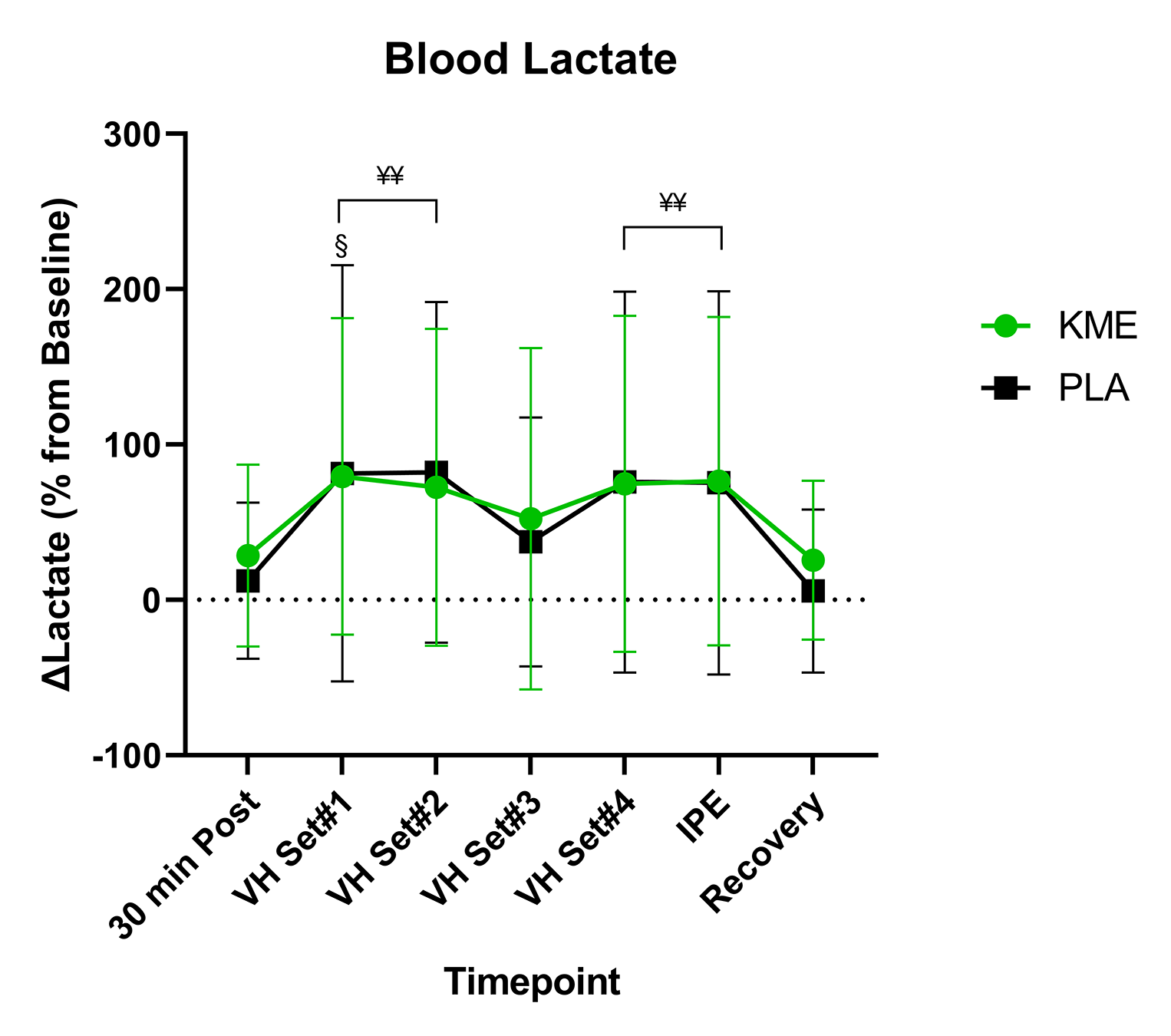

Supplement: Supplementary Figure 1 — Change in blood lactate. Change in blood lactate was compared to baseline with exogenous ketone monoester (KME) or calorie-controlled placebo (PLA). n = 15. Data: Mean ± SD. §p<0.05, significant difference between baseline and post-baseline timepoint in KME group. ¥¥p<0.01, significant difference between baseline and post-baseline timepoint in PLA group. VH, voluntary hypoventilation; IPE, immediately post-exercise. [file Image_1.tif]
